# Differential physiological responses of resistant and susceptible grape cultivars to Eutypa dieback

**DOI:** 10.1093/jxb/eraf103

**Published:** 2025-03-18

**Authors:** Gabriela C Sinclair, Renaud Travadon, Paula J Eschen, Christopher Wallis, Kendra Baumgartner, Chloé E L Delmas, Joshua F Hnizdor, Megan K Bartlett

**Affiliations:** Department of Viticulture & Enology, University of California, One Shields Avenue, Davis, CA 95616, USA; E2S UPPA, CNRS, IPREM, Université de Pau et des Pays de l’Adour, 64000 Pau, France; Department of Plant Pathology, University of California, One Shields Avenue, Davis, CA 95616, USA; United States Department of Agriculture-Agricultural Research Service, Crop Diseases, Pests and Genetics Research Unit, Parlier, CA 93648, USA; USDA-ARS, Davis, CA, USA; INRAE, BSA, ISVV, SAVE, 33882 Villenave d’Ornon, France; Department of Viticulture & Enology, University of California, One Shields Avenue, Davis, CA 95616, USA; Department of Viticulture & Enology, University of California, One Shields Avenue, Davis, CA 95616, USA; University of Birmingham, UK

**Keywords:** Disease resistance, Eutypa dieback, *Eutypa lata*, gas exchange, grapevine, grapevine trunk disease, pathogen detection, photosynthesis, viticulture

## Abstract

*Eutypa lata* is a fungal pathogen of grapevine that causes widespread economic damage and threatens vineyard longevity worldwide. This study was initiated to further understanding of how grapevines resist *E. lata* infections, using an integrated approach combining inoculation assays in the greenhouse with physiological and biochemical measurements. Resistant ‘Zinfandel’ and susceptible ‘Syrah’ grapevines were subjected to control and inoculation treatments, and assessed for gas exchange, water status, photosynthetic biochemistry, hydraulic conductivity, wood chemistry, and fungal spread (lesion length). Infection reduced leaf photochemical function and gas exchange in Zinfandel and increased these variables in Syrah (*P*<0.05). Infection produced shorter lesions in Zinfandel (*P*<0.05), suggesting that down-regulating gas exchange limited pathogen spread by reducing the carbon supply to the pathogen or fungal movement in the transpiration stream. Neither cultivar up-regulated wood defense compounds in response to infection, but proanthocyanidin and catechin levels were constitutively higher in Zinfandel, and stilbenoid and flavonoid contents were constitutively higher in Syrah (*P*<0.05). Altogether, this study is the first to show that, counterintuitively, down-regulating physiological function in response to infection improves long-term resistance to *E. lata.* Screening responses in photochemical function or gas exchange could provide a high-throughput alternative to measuring lesion lengths in assessing resistance.

## Teaser

Resistant ‘Zinfandel’ and susceptible ‘Syrah’ *Vitis vinifera* cultivars exhibited opposing responses in gas exchange and leaf photosynthetic biochemistry to fungal pathogen (*Eutypa lata* ) infection

## Introduction

*Eutypa lata*, the main causal agent of Eutypa dieback of grapevine and other perennial fruit and nut crops (Rolshausen *et al.*, 2015), is a pathogenic fungal ascomycete that reduces vineyard longevity and causes widespread economic damage to the viticultural sector (Munkvold *et al.*, 1994; Bertsch *et al.*, 2013; Kaplan *et al.*, 2016; Baumgartner *et al.*, 2019). Eutypa dieback is a major concern to growers due to the chronic nature of this disease that kills fruiting positions (Rolshausen *et al.*, 2008). Because *E. lata* is a wood-colonizing fungus that can form long-term necrotrophic relationships, infection can eventually kill the host (Rolshausen *et al.*, 2008). There is no cure for this complex disease, and detection is difficult, since the diagnostic foliar symptoms of Eutypa dieback may not appear until 3–8 years after the initial wood infection and these symptoms are not apparent consistently each year (Sosnowski *et al.*, 2007b, 2011; Etienne *et al.*, 2024; Travadon *et al.*, 2024). Eutypa dieback may also intensify in grape production areas where climate change intensifies rainfall events in the spring, potentially increasing spore production and inoculum pressure (Carter, 1991) and promoting foliar symptom expression (Sosnowski *et al.*, 2007b). Thus, identifying and enhancing the mechanisms underlying resistance to *E. lata* will be an important approach to mitigate the economic impacts of this disease (Cardot *et al.*, 2019).

Multiple studies have shown that grape cultivars vary in susceptibility to Eutypa dieback (Péros and Berger, 1994; Sosnowski *et al.*, 2007a; Travadon *et al.*, 2013, 2024; Moisy *et al.*, 2017; Sosnowski and Ayres, 2022; ; Sosnowski and Ayres, 2022; Travadon and Baumgartner, 2023; Etienne *et al.*, 2024). Grapevines are pruned during the dormant season, and most Eutypa infections occur when spores colonize the pruning wounds. Susceptibility to colonization, measured as the proportion of *E. lata* recovered from pruning wounds treated with inoculum, is similar between cultivars (Chapuis et al., 2007). Instead, variation in susceptibility to dieback is mainly determined by the rate of pathogen spread after colonization. Susceptibility to pathogen spread is typically measured from the incidence and severity of foliar symptoms, or the length of lesions formed in the wood by pathogen spread after inoculation (Sosnowski *et al.*, 2007a; Ramírez *et al.*, 2018; Travadon and Baumgartner, 2023).; Travadon and Baumgartner, 2023). The physiological strategies that make some cultivars more effective at halting wood colonization and minimizing foliar symptoms remain unclear. Previous work has shown that infected grapevines accumulate antifungal phenolic compounds in the wood around the infection site, and that resistant cultivars—those with less wood colonization (shorter lesions)—rapidly up-regulate genes related to defense compounds in response to infection (Rolshausen *et al.*, 2008; Cardot *et al.*, 2019; Galarneau *et al.*, 2021). However, *E. lata* impairs multiple physiological functions, suggesting that limiting this damage would also be an important resistance mechanism. Yet, to our knowledge, no studies have tested this hypothesis by comparing physiological responses to infection in cultivars of varying susceptibility to identify new *Eutypa* resistance mechanisms.

*Eutypa* impairs vine physiology in multiple ways. First, *E. lata* causes soft-rot wood decay. The mycelia release hydrolytic enzymes to break down cell walls in the stem and digest the glucose-rich cell wall components, and hydroxyl radicals and other toxins to induce cell death and facilitate wood decay (Rudelle *et al.*, 2005; Perez-Gonzalez *et al.*, 2022). Degrading woody tissues damages the xylem and stimulates the vine to block the xylem around the infection site with tyloses, to limit pathogen spread (Rudelle *et al.*, 2005). These processes are expected to reduce plant hydraulic conductivity and the capacity to supply water to the canopy, as observed for the Esca fungal complex (*Phaeomoniella chlamydospora*, *Phaeoacremonium minimum*, and *Fomitiporia* sp.) (Bortolami *et al.*, 2021). Second, fungal toxins and secondary metabolites travel to the leaves via the transpiration stream, where they are hypothesized to induce foliar symptoms (Octave et al., 2006, 2009, Andolfi *et al.*, 2011; Travadon and Baumgartner, 2023). The *E. lata* toxins (e.g. eutypine, eulatachromene, and benzofuran) accumulated in the leaf cytoplasm and damaged thylakoids and reduced leaf chlorophyll content (Amborabé *et al.*, 2001; Mahoney et al., 2003, 2005; Smith *et al.*, 2003; Octave et al., 2006; Sosnowski *et al.*, 2007; Andolfi *et al.*, 2011). Toxins secreted by the Esca complex reduced photosynthesis and chlorophyll fluorescence parameters (Petit *et al.*, 2006). However, the interactions between toxins and other fungal-secreted molecules and their contribution to leaf symptom severity and cultivar susceptibility remain unclear, especially since symptom expression is often inconsistent between vintages (Sosnowski *et al.*, 2007b; Travadon *et al.*, 2024).

Resistant cultivars have been hypothesized to have several characteristics that limit hydraulic and photochemical damage. First, the wood chemistry of resistant cultivars may be less conducive to colonization/decay, possibly due to a host response which includes generating a high concentration of antifungal phenolic compounds (e.g. resveratrol; Galarneau et al., 2025) and incorporating more lignin in the xylem (Rolshausen *et al.*, 2008; Lambert *et al.*, 2012; Pereira *et al.*, 2018). Lignin increases cell wall rigidity, which can slow pathogen growth and wood decay (Pereira *et al.*, 2018). Lesion lengths were shorter in a cultivar with higher xylem lignin content (i.e. ‘Merlot’ compared witho ‘Cabernet Sauvignon’) (Rolshausen *et al.*, 2008). Second, resistant cultivars could have more effective leaf detoxification mechanisms (Legrand *et al.*, 2003; Legrand *et al.*, 2003; Andolfi *et al.*, 2011). In resistant, but not susceptible, cultivars *E*. *lata* infection stimulated the leaves to rapidly up-regulate many defense-related genes, including genes controlling phenolic pathways (Cardot *et al.*, 2019). Resistant cultivars could also counteract phytotoxicity by accumulating solutes that prevent cellular damage through antioxidant activity (i.e. osmoprotectants), as part of osmotic adjustment, though this mechanism has not previously been tested (Patakas et al., 2002; Zivcak *et al.*, 2016). Finally, photochemical and hydraulic damage could have downstream effects on gas exchange that impact pathogen spread and resistance. Impaired hydraulic or photochemical function could lead vines to close their stomata and reduce gas exchange to avoid placing additional stress on the hydraulic system or declines in water use efficiency (WUE; Sosnowski *et al.*, 2011; Pouzoulet *et al.*, 2014). This response could increase susceptibility by reducing the plant carbon supply for costly defense compounds, or benefit resistance by limiting toxin and pathogen movement in the transpiration stream and the photoassimilate available to the pathogen. These competing hypotheses have not been tested. Collectively, understanding the role of these mechanisms in infection responses will offer new insights into how grapevines respond to *E*. *lata* and resist pathogen attack in the early stages of infection.

We conducted the first study to address how physiological responses to infection are associated with cultivar susceptibility to *E. lata* infection. We measured the effects of infection on vine physiology—including gas exchange, photochemical function, hydraulics, and osmotic adjustment—and wood chemistry profiles for two cultivars (*Vitis vinifera* ‘Zinfandel’ and ‘Syrah’) that differ in resistance. Zinfandel has been classified as resistant (Travadon and Baumgartner, 2023) and Syrah as susceptible (Loschiavo *et al.*, 2007; Sosnowski *et al.*, 2007a) to Eutypa dieback based on wood lesions and leaf symptoms. These cultivars also differ in their physiology. Syrah has higher gas exchange rates than Zinfandel, whereas Zinfandel has more negative leaf osmotic potentials, which could indicate a higher concentration of osmoprotectant compounds (Charrier *et al.*, 2018; Gallo *et al.*, 2021; Sinclair *et al.*, 2024). We hypothesized that infected Zinfandel would maintain greater leaf photochemical function than infected Syrah, in part because infection would up-regulate osmotic adjustment for Zinfandel. We also expected that infected Zinfandel would more strongly up-regulate wood defense compounds, which would reduce lesion spread and declines in plant hydraulic conductance compared with infected Syrah. Together, we expected this combination of lower photochemical and hydraulic damage to allow infected Zinfandel to maintain greater gas exchange than infected Syrah. For each cultivar, we compared wounded, non-inoculated controls with vines inoculated with *E. lata* isolate BX1-10, originally isolated from Bordeaux, France, which is a virulent isolate used in phenotyping for resistance to Eutypa dieback (Moisy *et al.*, 2017; Travadon *et al.*, 2023; Travadon *et al.*, 2023). We maintained vines under well-watered conditions to avoid confounding effects of drought on fungal colonization, and instead focus on the effects of infection on host physiology. We tested whether the physiology variables, lesion length, and wood chemistry varied between the two cultivars and inoculation treatments using an integrated approach combining classic pathogenicity assays, molecular detection of the pathogen, and physiological and biochemical measurements. Altogether, we expected this study to advance our understanding of the physiological responses at the chemical, cellular, and whole-plant level that promote resistance to *E. lata* in the early stages of this chronic disease. Identifying important traits could also improve screening for disease resistance and aid the development of more resilient plant material.

## Materials and methods

### Plant material and growth conditions

*Vitis vinifera* cultivars Zinfandel and Syrah were propagated from dormant, certified disease-free cuttings in March in Davis, CA, USA. The certification process tests for 38 diseases, including 36 viruses (https://fps.ucdavis.edu/fgr2010.cfm). The plant material was provided by the University of California, Davis Foundation Plant Services. Cuttings were callused in a dark, humidified room in containers layered with equal parts vermiculite and perlite for 2–3 weeks. Once rooted, the cuttings were planted in paper carton inserts containing a mixture of sunshine mix, perlite, and vermiculite, and allowed to establish in the same room for an additional 2 weeks. Afterwards, the plants were transitioned to an auto-controlled mist room for 2 weeks to acclimate, then transferred to a greenhouse. Plants were subsequently transplanted to 1 gallon (4.54 liter) pots containing an agronomy mix (60:40 Agromix, perlite soil mixture) with an extended-release fertilizer, and well-watered to field capacity weekly until the experimental period.

### *Eutypa lata* (isolate BX-10) inoculation

During the winter prior to experimentation (6 January), each cultivar was divided into two categories: non-inoculated wounded (NIW) controls (*n*=5) and inoculated wounded (INOC) (*n*=22–25). All plants were pruned to three buds and inoculated following procedures outlined by Travadon *et al.* (2013). In brief, a 4.4 mm width×3 mm depth injection site was created 2 cm directly below the main upper node on each vine cutting, using a 4.4 mm power drill to penetrate the woody stem ([Bibr CIT0064][Bibr CIT0064]). The plants designated for the inoculated treatments were then inoculated with mycelium plugs (4.2 mm diameter) taken from the margin of a 5-day-old culture of *E. lata* isolate BX1-10 on potato dextrose agar (PDA; Difco, Detroit, MI, USA). The plants designated for the NIW treatment were mock-inoculated with a sterile PDA plug of equal size. The wound was sealed with Vaseline (Unilev er, Greenwich, CT, USA) and wrapped with Parafilm (American National Can, Chicago, IL, USA). Following inoculation, the plants were left for 1 week in the greenhouse to establish infection and then moved to the lathe house for 5 months. On 10 June, the plants were transferred back into the greenhouse and transplanted to 2.5 gallon (11.36 liter) pots containing agronomy mix and extended-release fertilizer.

### Watering regime

Saturated weight (SW) for each pot was established by watering pots to field capacity, waiting until dripping from the bottom ceased (~2 h), then recording the weight. All pots were maintained at well-watered conditions for 2 weeks before the start of the experiment to allow for acclimatization by re-watering to SW every 3 d. During the experiment, the pots were weighed and re-watered to target weights, following the methods from Bartlett et al. (2021). Initially, pots were re-watered every Monday, Wednesday, and Friday to a target weight of 95% of SW plus half of the expected pot evapotranspiration between waterings, which was calculated from the change in pot weights during the acclimation period (Bartlett et al., 2021; Pita and Pardos, 2001). The pots were watered following this regime until August, when heat waves forced us to increase the frequency of watering to 5 d a week to avoid water stress. Watering treatments were maintained until 8 September, the date of the destructive harvest. During the experiment, the pot surface was covered with aluminum foil to limit evaporation from the soil. Greenhouse environmental conditions [temperature, relative humidity, and vapor pressure deficit (VPD)] were tracked throughout the experiment (Supplementary Figs S1, S2).

### Gas exchange, water potential, and plant hydraulic conductivity

We measured gas exchange on one fully expanded, mature asymptomatic leaf per vine, at positions 6–12 leaves below the growing tip, weekly from 8 July 8 to 2 September with a portable gas exchange system (Li-Cor 6800 Portable Photosynthesis System, Lincoln, NE, USA). Gas exchange parameters include a fan speed of 10 000 rpm, CO_2_ concentration level of 400 µmol mol^–1^, light intensity of 1200 µmol m^–2^ s^–1^, and VPD set point of 1.8 kPa. The temperature set point was ~30±2 °C at the beginning of the experiment (July) and 32±2 °C over the course of the hotter months of the experiment (August–September). We allowed humidity in the sample chamber to match ambient conditions.

Pre-dawn (04.00 h to 06.00 h) and midday (11.00 h to 16.00 h) stem water potentials [pre-dawn and midday stem water potential (PDLWP and MDSWP)] were measured on the same day as gas exchange on one fully expanded, mature leaf per vine using a pressure chamber (PMS Instrument; model 1505D). The leaves were acclimated in dark-adapting bags for 30 min prior to excision, then measured immediately or stored in humidified Whirl-Pak bags in a refrigerator for up to 3 d before measuring. Whole-plant evapotranspiration (*E*_tot_) was calculated from the change in pot weight between watering intervals, normalized by the canopy area, and whole-plant hydraulic conductance (*K*_plant_) was calculated as *K*_plant_=*E*_tot_/(PDLWP−MDSWP).

### Leaf chlorophyll concentration and fluorescence

At two time points (12 August and 2 September) at midday, two fully expanded, asymptomatic leaves per vine from the same positions (6–12 from the growing tip) were measured for chlorophyll content using a chlorophyll concentration meter (Apogee MC-100, UT, USA). The same leaves were then dark adapted for 30 min inside dark-adapting bags to fully open PSII reaction centers and, immediately before taking the chlorophyll fluorescence measurement, placed inside a tent made of black plastic bags to limit sun exposure. The settings for determining *F*_v_/*F*_m_ were outlined in the user instructions in the LICOR-6800 manual. The actinic light was turned off and the measuring beam turned on with a dark mode rate set to 50 Hz. The rectangular flash had a red target set to 8000 μmol m^–2^ s^–1^ and a duration of 1000 ms. The leaves were then excised to measure midday stem water potential.

### Leaf osmotic potential

Leaf osmotic potentials at full turgor (π_o_) were measured for each vine 6 and 8 months after inoculation (12 July and 6 September). We sampled one asymptomatic, fully expanded leaf per vine and recut the petiole under water using a fresh razor blade. We then rehydrated the leaves overnight in tubes of deionized water. Leaf hydration was standardized by beginning and ending rehydration for all leaves at the same time and storing the leaves in humidified Whirl-Pak bags in a refrigerator until measuring. We measured leaf osmotic potential following the rapid osmometer method from Bartlett et al. (2012) using a vapor pressure osmometer (Vapro 5520, Wescor, Logan, UT, USA).

### Destructive harvest

On 8 September, canopies and shoots were excised from the main stem from all plants. Five canopies per cultivar per treatment were measured for total leaf area. The root biomass was carefully removed from the main stem, shaken to remove excess soil, and rinsed before drying in a drying oven at 114 °C for a few weeks. The stem of the inoculated and non-inoculated cuttings was used to measure lesion length, pathogen recovery, and wood chemistry. We measured lesion length in the inoculated internode. The bark was scraped from their woody stems and the stem was cut lengthwise through the inoculation site to allow measuring the internal lesion length (up and down from the inoculation site) using digital calipers. One half of the stem was stored at –80 °C and used for molecular detection of the pathogen. The other half of the stem was flash-frozen in liquid nitrogen and kept at –80 °C for wood chemistry.

### DNA-based detection of *E*. *lata* using quantitative PCR

Due to low and inconsistent recovery rates of *E*. *lata* using culture-based methods, we adopted a DNA-based detection approach previously used for detecting the pathogen from grapevine wood (Brown et al., 2020; Travadon and Baumgartner, 2023). This approach relies on adapting quantitative PCR (qPCR) procedures (Pouzoulet *et al.*, 2013, 2017) to a qualitative assay, as outlined by Brown et al. (2021). On the half stem used for pathogen detection, a 3 cm section encompassing the inoculation site (1.5 cm above and below) of wood was collected for grinding. DNA was extracted from 100 mg of cryogenically ground wood tissue following tissue grinding procedures outlined by Galarneau *et al.* (2021). The exact DNA extraction procedure, including extraction buffers and kit used, and qPCR conditions have been detailed by Brown et al. (2021) and Baumgartner et al. (2023). After the PCR amplifications were completed, dissociation curves were obtained. Genomic DNA from pure cultures was used as a positive control. Amplification of target DNA was based on the dissociation temperature (79.0–79.5 °C for *E*. *lata*). Positive detections were samples crossing the threshold level by 45 cycles.

### Wood chemistry

Total phenolics and lignin content was measured using extraction methods by Wallis *et al.* (2012). In brief, 100 mg of cryogenically ground wood tissue was added to 500 μl of methanol (LC-MS grade, Fisher Scientific, Pittsburgh, PA, USA), vortexed, and left overnight on a shaker in a cold room (4 °C). The following day, the samples were centrifuged at 10 000 *g* for 1 min, the supernatant was removed, and the previous steps were repeated to re-extract the pellet. The next day, both supernatants were combined for a final volume of 1 ml. The tubes were centrifuged briefly, and 150 μl of the supernatant was placed into 2 ml glass vials with glass inserts.

HPLC was conducted using a Shimadzu (Columbia, MD, USA) LC-20AD-based system equipped with a Supelco Ascentis C18 reverse-phase column (Millipore-Sigma, St. Louis, MO, USA) and a photodiode array detector for quantification (with peak areas obtained at 280 nm) (Wallis *et al.*, 2012). For each sample, 50 µl were injected, with a binary gradient proceeding from 0.2% (v/v) acetic acid in water to 0.2% (v/v) acetic acid in methanol over a 40 min run (Wallis *et al.*, 2012). Compound identification and quantification were carried out by matching retention times with standards obtained from Millipore-Sigma, and based on previous compound identifications by Wallis *et al.* (2012). Levels of individual compounds within a phenolic subclassification (stilbenoids, proanthocyanidins/catechins, or other flavonoids) were summed together for analyses, as well as an overall sum of all quantified phenolic compounds.

Lignin extraction was completed using the same pellet for the phenolic extractions. All reagents were obtained from Millipore-Sigma. First, the leftover pellet was washed with 1 ml of ultra-pure H_2_O. Then 800 µl of 2 N HCl was pipetted onto the pellet followed by 300 µl of mercaptoacetic acid. This solution was incubated at 86 °C for 4 h. Afterwards, the supernatant was discarded and the pellet was washed twice with 1 ml of water. Next, 1 ml of 0.5 M NaOH was added to the sample, and the supernatant and pellet were mixed then placed on a vortexer shaker overnight. The next day, the tubes were centrifuged at 10 000 *g* for 2 min and the supernatant was removed and saved at 4 °C. With the remaining pellet, 500 µl of 0.5 M NaOH was added and the vortexer shaking overnight and centrifuge steps were repeated once more. Both supernatants from the overnight steps were combined with 300 µl of concentrated HCl, vortexed, and incubated at room temperature for 4 h. Afterwards, the supernatant samples were centrifuged for 2 min at 10 000 *g*. The supernatant was then discarded, and the remaining pellet precipitated from the supernatants combined with 300 µl of concentrated HCl was dried overnight in a fume hood. The following day, the pellet was mixed with 1 ml of 0.5 M NaOH and allowed to rest at room temperature for 4 h. Finally, 1 µl aliquots of this solution were diluted in 99 µl of 0.5 M NaOH and used to measure lignin absorbance at 280 nm utilizing a microplate reader. Lignin concentrations were calculated from a standard curve spanning 0, 18, 45, 90, and 180 µg ml^–1^.

### Statistical analysis

All analyses were performed in Rstudio (R version 4.2.2). First, Shapiro tests were performed to confirm the data were normally distributed (*P*>0.05 for all variables). We then used type III ANOVA to test the main and interactive effects of inoculation (Treatment), cultivar (Cultivar), and number of days after inoculation (Timepoint), on stomatal conductance (*g*_s_), net photosynthesis (*A*), leaf transpiration (*E*), WUE, PDLWP, MDSWP, and whole-plant hydraulic conductivity (*K*_plant_). We included Timepoint and the interaction with Treatment as predictors to test whether inoculation effects became stronger over time. A type III ANOVA was also used to test whether differences in gas exchange were impacted by differences in soil water availability by using PDLWP, inoculation (Treatment), cultivar (Cultivar), number of days after inoculation (Timepoint), and their interactions as predictors. We represented sampling date as a categorical variable (Date) in analyses of osmotic potential at full hydration (OSM), chlorophyll content, and maximum quantum yield of PSII (*F*_v_/*F*_m_), since these variables were measured twice (i.e. OSM~Cultivar+Treatment+Date+Cultivar×Treatment+Date×Treatment). Analyses of lesion length, root biomass, leaf canopy area, and wood chemical composition excluded time, as these variables were measured once at the end of the experiment. Differences between statistically significant effects were evaluated and further compared with post-hoc Tukey’s HSD tests. We also repeated these analyses using the subset of inoculated vines where the establishment of *E. lata* was confirmed with qPCR detection and generally found the same results, with no significant changes in the results for photosynthesis, chlorophyll content, *F*_v_/*F*_m_, osmotic adjustment, and wood chemistry (see the Results ‘Inoculation treatments’ and Supplementary Data Tables S1–S5.

## Results

### Inoculation treatments: pathogen detection, stem lesions, root biomass and canopy leaf area

*Eutypa lata* DNA was not detected in any of the NIW plants, indicating the absence of the pathogen in the control grapevine stems. Conversely, *E*. *lata* DNA was positively detected in 31 of the 42 (74%) inoculated plants, hence suggesting the successful establishment of the pathogen in inoculated plants.

**Fig. 1.**
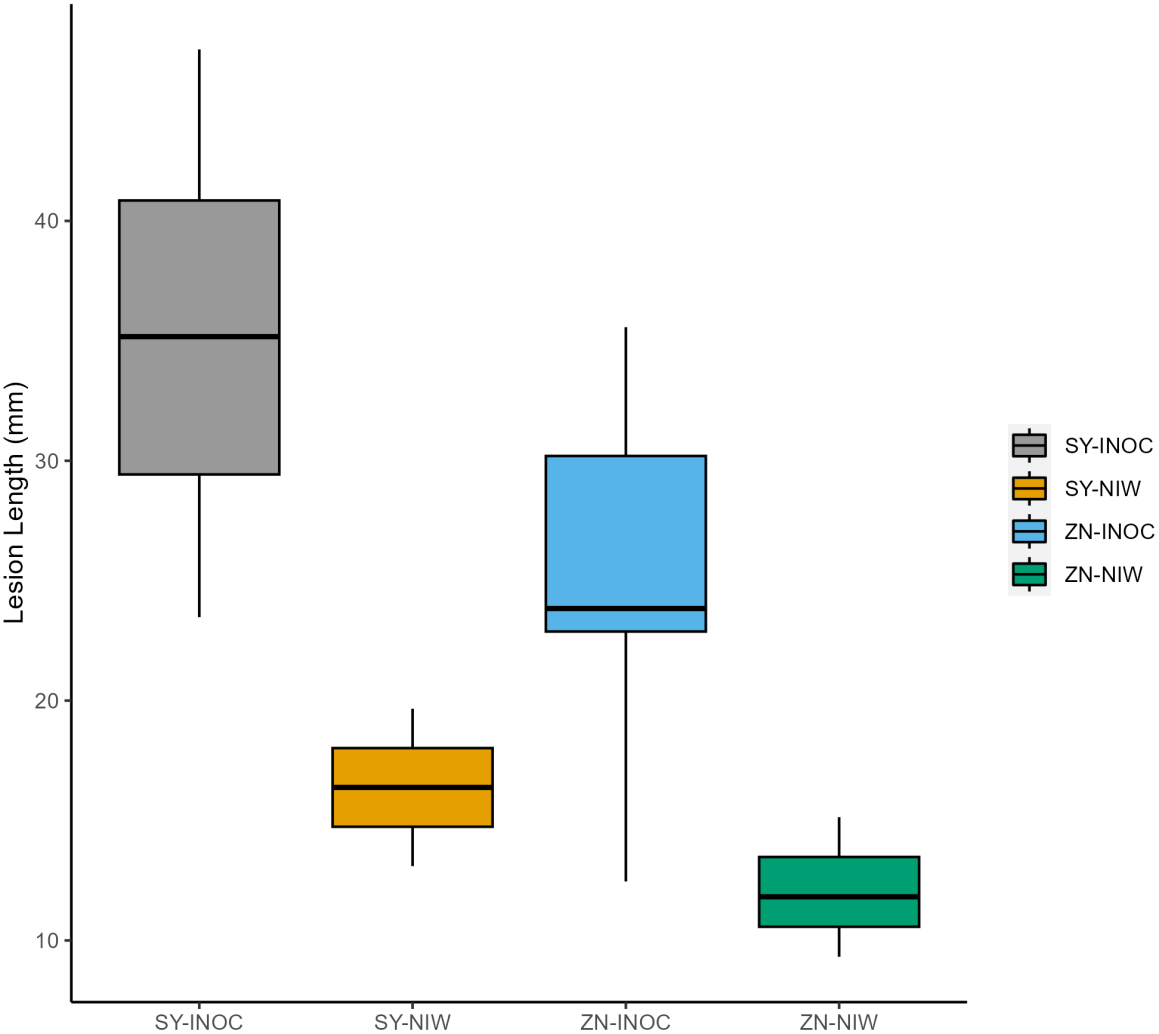
Internal wood lesion length of each cultivar and treatment measured in millimeters. Syrah (SY) had significantly higher lesion lengths than Zinfandel (ZN) treatments. Box plots represent averages of Syrah and Zinfandel inoculated plants (INOC) (*n*=22–25) and non-inoculated wounded control plants (NIW) (*n*=5).

We found significant effects of Cultivar (ANOVA *P*-value <0.0001) and Treatment (ANOVA *P*-value <0.001) on lesion length. Total lesion length was significantly higher in inoculated Syrah (35.19±1.48 mm) than inoculated Zinfandel (25.13±1.38 mm). Lesions were also present in NIW plants of both cultivars but were significantly smaller than those of inoculated plants (Table 1; Fig. 1) and did not test positive for *E. lata* DNA.

**Table 1.** Type III ANOVA results for variables measured once at the end of the experimental period

Predictor	Lesion length	Root biomass	Canopy leaf area
Cultivar	7e-06***	0.3 NS	1e-03**
Treatment	3e-04***	0.7 NS	0.8 NS
Cultivar×Treatment	0.4 NS	0.6 NS	0.9 NS

**P<0.01, ***P<0.001. NS represents non-significant results.

Root biomass was not statistically different between Cultivars, Treatments, or their interactions. Conversely, total canopy leaf area was significantly different between Cultivars, with Syrah exhibiting a larger canopy area (Supplementary Table S6). Total canopy leaf area was not significantly affected by Treatment or Cultivar×Treatment interactions (ANOVA *P*-value >0.05; Table 1).

### Plant gas exchange, water status, and hydraulics

**Fig. 2.**
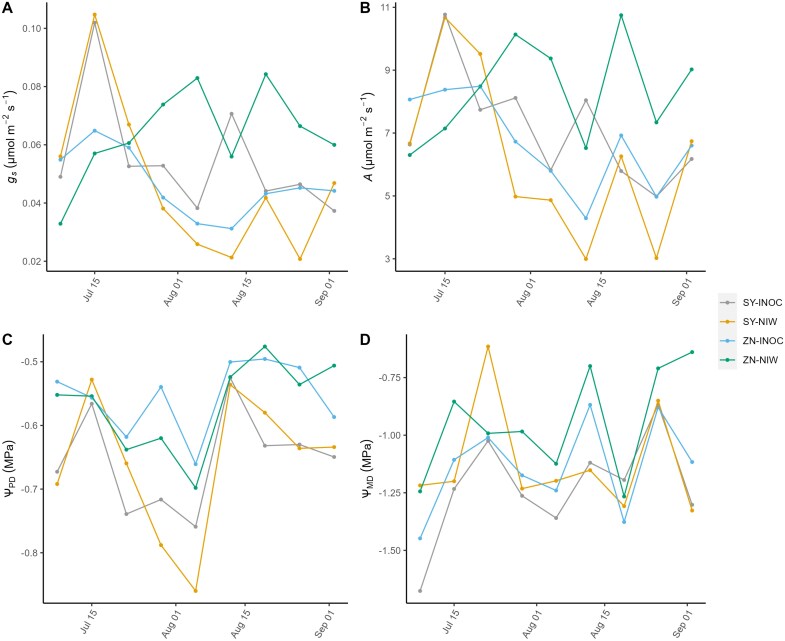
Gas exchange, and pre-dawn leaf and midday stem water potential for each cultivar and treatment over the experimental period. The *x*-axis contains sampling dates: 15 July (Jul 15), 1 August (Aug 01), 15 August (Aug 15), and 1 September (Sep 01). Data points represent averages of Syrah (SY) and Zinfandel (ZN) inoculated plants (INOC) (*n*=22–25) and non-inoculated wounded control plants (NIW) (*n*=5).

There were significant interactive effects of Cultivar and Treatment on all gas exchange variables except WUE, including *g*_*s*_, *E*, and *A* (Table 2). These variables also varied significantly during the experiment, though the interaction between Timepoint and Treatment was not significant for *g*_s_, *A*, and *E*, indicating that the time effect was independent of inoculation treatment (Fig. 2A, B; Supplementary Fig. S3). Conversely, for whole-plant evapotranspiration (*E*_tot_), we found a significant interactive effect of Treatment×Timepoint on *E*_tot_ as well as significant main effects of Cultivar, Timepoint, and Treatment (Table 2; Supplementary Fig. S4B).

**Table 2.** Type III ANOVA results for variables measured repeatedly during the experiment

Predictor	*g*_*s*_	*A*	*E*	WUE	PDLWP	MDSWP	*E*_tot_	*K*_plant_
Cultivar	5e-03**	0.1 NS	0.03*	0.4 NS	1e-13***	2e-16***	2e-16***	0.9 NS
Treatment	0.08 NS	8e-03**	0.09 NS	0.4 NS	0.6 NS	4e-03**	0.03*	0.4 NS
Timepoint	8e-07***	4e-12***	2e-03**	0.01*	0.09 NS	0.06 NS	2e-16***	0.03*
Cultivar×Treatment	5e-04***	7e-05***	1e-03*	0.9 NS	0.8 NS	0.2 NS	0.2 NS	0.9 NS
Treatment×Timepoint	0.3 NS	0.07 NS	0.3 NS	0.9 NS	0.5 NS	0.3 NS	0.01*	0.1 NS

*g*_s_, Stomatal conductance; *A*, photosynthesis; *E*, leaf-level transpiration; WUE, water use efficiency; PDLWP, pre-dawn stem water potential; MDSWP, midday stem water potential; *E*_tot_, whole-plant evapotranspiration; *K*_plant_, whole-plant hydraulic conductivity. Predictor variables are Cultivar, Treatment (inoculated versus wounded controls), Timepoint (days since the start of the experiment), and their interactions (Cultivar×Treatment and Treatment×Timepoint). Asterisks represent significance (*P<0.05, **P<0.01, ***P<0.001). NS represents non-significant results.

Relative to non-inoculated plants, plants inoculated with the pathogen had lower photosynthesis and lower stomatal conductance in Zinfandel but higher photosynthesis and higher stomatal conductance in Syrah. Mean *g*_s_ (averaged across time points) for Zinfandel was 0.064±0.004 mol m^–2^ s^–1^ (mean ±SE) for the NIW treatment and 0.046±0.002 mol m^–2^ s^–1^ for the INOC treatment (Table 3). Mean *A* (averaged across time points) for Zinfandel was 8.34±0.4 µmol m^–2^ s^–1^ and 6.72±0.21 µmol m^–2^ s^–1^ for the NIW and INOC treatments, respectively (Table 3). Conversely, mean *g*_s_ and *A* were higher in the inoculated treatment for Syrah (0.055±0.004 mol m^–2^ s^–1^ and 7.12±0.19 µmol m^–2^ s^–1^, respectively) than in the NIW treatment (0.047±0.005 mol m^–2^ s^–1^ and 6.19±0.46 µmol m^–2^ s^–1^) (Table 2; Fig. 2A, B).

**Table 3.** Cultivar and treatment means for variables

Treatment	*g*_s_ (mol m^–2^ s^–1^)	*A* (µmol m^–2^ s^–1^)	PDLWP (MPa)	MDSWP (MPa)	*K*_plant_ (kg MPa^–1^ s^–1^ m^–2^)	π_o_ (MPa)	Chl (µmol m^–2^)	*F*_v_ */F*_m_ (–)	Lesion length (mm)
Zinfandel (NIW)	0.064±0.004 a	8.34±0.40 a	–0.57±0.02 a	–0.95±0.06 a	0.0012±1.4e-04 a	–1.48±0.04 a	18.00 ±1.18 a	0.789±0.007 a	12.09±1.69 c
Syrah (NIW)	0.047±0.005 bc	6.19±0.46 b	–0.66±0.02 b	–1.14±0.05 b	0.0011±9.5e-05 a	–1.68±0.14 a	12.61±0.81 b	0.758±0.009 b	16.38±3.28 bc
Zinfandel (INOC)	0.046±0.002 c	6.72±0.21 b	–0.56±0.01 a	–1.14±0.02 b	0.0010±4.0e-05 a	–1.61±0.04 a	17.49±0.55 a	0.766±0.004 ab	25.13±1.38 b
Syrah (INOC)	0.055±0.004 ab	7.12±0.19 b	–0.65±0.01 b	–1.23±0.02 b	0.0011±4.2e-5 a	–1.65±0.03 a	14.22±0.39 b	0.773±0.004 ab	35.19±1.48 a

*g*_s_, Stomatal conductance; *A*, photosynthesis; PDLWP, pre-dawn stem water potential; MDSWP, midday stem water potential; *K*_plant_, whole-plant hydraulic conductivity; π_o_, leaf osmotic potential at full hydration; Chl, leaf chlorophyll content; *F*_v_/*F*_m_, quantum efficiency of PSII. Values are means ±SEs. Letters show Tukey HSD test results for significant main effects (ANOVA, *P*-value <0.05). *n*=22–25 for inoculated (INOC) Syrah and Zinfandel and *n*=5 for non-inoculated wounded (NIW) Syrah and Zinfandel for all variables except *F*_v_/*F*_m_, where *n*=5 for all cultivar and treatment combinations.

PDWP varied significantly between cultivars (ANOVA *P*-value <0.0001; Table 2; Fig 2C) and this was the only significant fixed effect on PDWP. There was a significant effect of PDWP on gas exchange (specifically on *g*_s_ and *E*; Table 4). However, when controlling for PDWP in our statistical model, there was still a significant Cultivar×Treatment impact on gas exchange, specifically on *g*_s_, *A*, and *E*, indicating that this interactive effect is not just due to accidental variation in water availability (ANOVA *P*-value <0.001; Table 4).

**Table 4.** Type III ANOVAs testing whether cultivar and treatment differences in gas exchange are driven by differences in pre-dawn leaf water potential (PDLWP)

Predictor	*g*_s_	*A*	*E*	WUE
Cultivar	0.02*	0.3 NS	0.1 NS	0.6 NS
Treatment	0.3 NS	0.4 NS	0.3 NS	0.9 NS
Timepoint	2e-07***	4e-12***	0.001**	0.01*
PDLWP	6e-04***	0.07 NS	0.002**	0.06 NS
Cultivar×Treatment	4e-04***	1e-04***	0.001**	1 NS
Treatment×PDLWP	0.5 NS	0.7 NS	0.5 NS	0.9 NS
Cultivar× PDLWP	0.2 NS	0.6 NS	0.4 NS	0.9 NS

Abbreviations follow Table 2. Cultivar and treatment interaction effects remained significant for *g*_s_, *A*, and *E* and non-significant for WUE, indicating that cultivar differences in soil water status did not explain the cultivar and treatment differences in gas exchange. *P<0.05, **P<0.01, ***P<0.001. NS represents non-significant results.

On the other hand, there was a significant effect of Cultivar and Treatment on midday stem water potential (ANOVA *P*-value <0.005, Table 2). Inoculated Zinfandel and Syrah both had more negative mean midday stem water potentials than the non-inoculated controls (Fig. 2D).

Finally, there was no significant effect of any of the predictors on whole-plant hydraulic conductivity except Timepoint (Table 2), indicating that the changes in hydraulic conductivity over time are likely to be in response to increased water demand from canopy development and climate-related variables (Supplementary Figs S1, S2).

### Leaf chlorophyll and fluorescence measurements

**Fig. 3.**
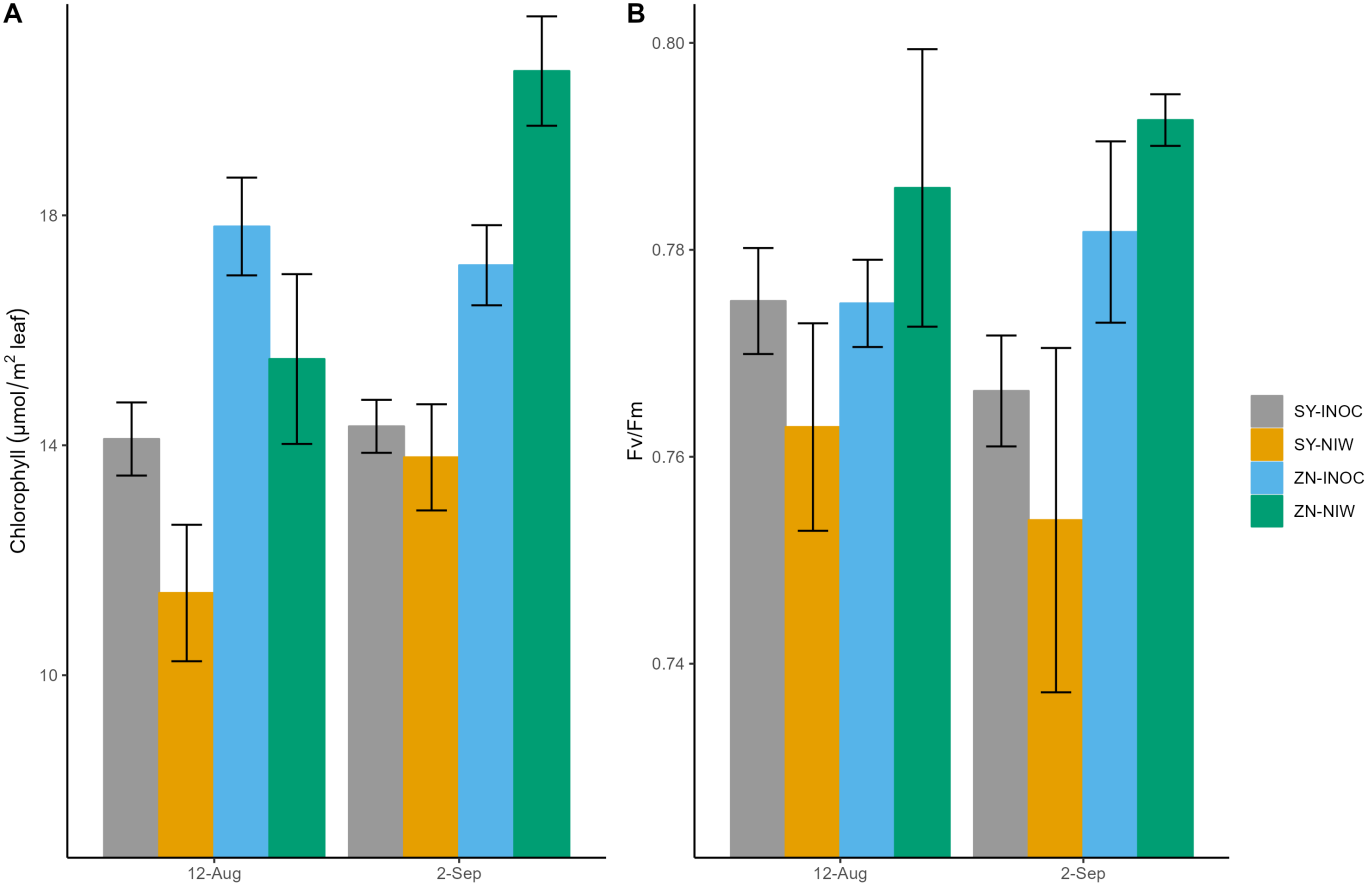
Mean leaf chlorophyll concentration and fluorescence values. (A) Mean leaf chlorophyll concentration in µmol m^–2^ of leaf tissue. Data represent averages for the two sampling dates [6 and 8 months post-inoculation: 12 August (12-Aug) and 2 September (2-Sep)] per cultivar and treatment. Bar graphs for the first sampling date represent averages of inoculated (INOC) (*n*=22–25) and non-inoculated wounded control plants (NIW) (*n*=5). The second sampling date represents averages of INOC (*n*=5) and NIW plants (*n*=5). Chlorophyll content significantly differed between Cultivar and Treatment. (B) Fluorescence values (*F*_v_*/F*_m_) taken on the same sampling date. There were significant interaction effects of Cultivar and Treatment on *F*_v_*/F*_m_. SY stands for Syrah and -INOC refers to the inoculated treatment group. ZN stands for Zinfandel and -INOC refers to the inoculated treatment group. The mock-inoculated control group is labeled NIW.

There was a significant interactive effect of Treatment×Date on chlorophyll content, and a significant interactive effect of Cultivar×Treatment on chlorophyll fluorescence (Table 5). *F*_v_/*F*_m_ and chlorophyll content were higher in inoculated (14.22±0.39 and 0.773±0.004 µmol m^–2^ of leaf, respectively) than in non-inoculated (NIW) vines for Syrah (12.61±0.81 and 0.758±0.009 µmol m^–2^), while they were lower in inoculated (17.49±0.55 and 0.776±0.004 µmol m^–2^) than in non-inoculated vines (18.0±1.18 and 0.789±0.007 µmol m^–2^) for Zinfandel (Table 3; Fig. 3).

**Table 5.** Type III ANOVA results for variables measured twice during the experiment

Predictor	π_o_	Chl	*F*_v_ */F*_m_
Cultivar	0.5 NS	4e-06***	0.7 NS
Treatment	0.7 NS	0.01*	0.2 NS
Date	2e-05***	0.7 NS	0.9 NS
Cultivar × Treatment	0.1 NS	0.2 NS	0.02*
Treatment × Date	0.3 NS	0.01*	1 NS

Since these variables were measured less often, time since the start of the experiment is represented with the categorical variable Date instead of the continuous variable Timepoint (Table 2). *P<0.05, ***P< 0.001, and NS for non-significant results.

π_o_, Leaf osmotic potential at full hydration; Chl, leaf chlorophyll content; *F*_v_/*F*_m_, quantum efficiency of PSII.

### Osmotic potential

There was no significant effect of any of the predictors on osmotic potential except Date (ANOVA *P*-value <0.001, Table 5), indicating osmotic adjustment between the two sampling periods, but independently of the impacts of infection.

### Wood chemical composition

Among the different fixed effects, only ‘Cultivar’ had a significant effect on total (ANOVA *P*-value <0.008; Table 6) or individual chemical compounds (Supplementary Table S7). Proanthocyanidins (catechins) were significantly higher in Zinfandel than in Syrah (Table 7). Total stilbenoid and other flavonoid concentrations—derived from total phenolic extractions—were higher in Syrah in comparison with Zinfandel. Lignin content and overall phenolic concentrations were not significantly different between treatments or cultivars (ANOVA *P*-value >0.2; Table 7).

**Table 6.** Type III ANOVA results for wood chemistry measured at the end of the experimental period

Predictor	Lignin	Total phenolics	Total procyanidins/catechins	Total stilbenoids	Total flavonoids
Cultivar	0.8 NS	0.2 NS	6e-05***	7e-03***	9e-06***
Treatment	0.4 NS	0.8 NS	1 NS	0.4 NS	0.7 NS
Cultivar×Treatment	0.4 NS	0.9 NS	0.9 NS	1 NS	0.8 NS

***P<0.001. NS represents non-significant results.

**Table 7.** Wood chemical composition (mg g^–1^ FW) for each cultivar and treatment

Treatment	Lignin	Total phenolics	Total procyanidins/catechins	Total stilbenoids	Total other flavonoids
**Zinfandel (NIW)**	17.44±0.14 a	31.53±7.3 a	23.86±5.94 a	3.07±0.82 ab	4.57±1.47 ab
**Syrah (NIW)**	17.32±0.05 a	26.43±3.8a	13.36±1.96 b	4.09±0.47 a	8.96±1.96 a
**Zinfandel (INOC)**	17.39±0.04 a	29.33±1.60 a	23.55±1.89 a	2.54±0.23 ab	3.22±0.29 b
**Syrah (INOC)**	17.41±0.04 a	25.44±2.07 a	13.56±0.94 b	3.57±0.27 a	8.28±0.95 a

Values are means ±SE for wood near the inoculation site. *n*=22–25 for inoculated (INOC) Syrah and Zinfandel, and *n*=5 for non-inoculated wounded control (NIW) Syrah and Zinfandel. There were significant cultivar differences in chemistry, but no effects of treatment or interactive effects between treatment and cultivar. Letters show Tukey HSD test results.

## Discussion

This study is the first to test how physiological responses to infection vary with resistance to Eutypa dieback. We found that the resistant cultivar Zinfandel down-regulated physiological function in response to infection, while the susceptible cultivar Syrah maintained or even improved function, suggesting that there are trade-offs between short-term performance and long-term resistance to this slow-acting disease. Zinfandel and Syrah have been classified as resistant and susceptible based on lesion spread in the wood of inoculated vines (Travadon and Baumgartner, 2023), and our findings support this classification, with infection associated with longer lesions in Syrah than in Zinfandel (Fig. 1). Contrary to our hypotheses, infection was associated with greater leaf photochemical damage in Zinfandel, which would reduce photosynthetic capacity and induce stomatal closure, producing the observed declines in gas exchange (Figs 2, 3). In contrast, photochemical function and gas exchange increased in infected Syrah, suggesting that Syrah was less sensitive to fungal toxins than Zinfandel and instead up-regulated leaf photochemistry and gas exchange, potentially to improve the carbon supply for fungal defense. This strategy did not produce hydraulic damage in infected Syrah under the well-watered conditions in this study (Table 2), but may have promoted pathogen growth by increasing fungal movement in the transpiration stream or the carbon and nutrient supply to the pathogen. This strategy also did not improve the production of wood phenolic compounds, as proanthocyanidin/catechin levels were constitutively higher in Zinfandel, and stilbenoid and flavonoid contents were constitutively higher in Syrah and were not up-regulated in response to infection (Tables 6, 7). Altogether, these findings show that vine performance responses to infection can have counterintuitive effects on long-term resistance, with worse impacts of infection on photochemistry and gas exchange seemingly helping the resistant cultivar reduce pathogen growth and lesion spread. These findings also suggest that screening for infection responses in chlorophyll content and fluorescence or *g*_s_ could provide a high-throughput alternative to measuring lesion lengths in breeding programs for Eutypa resistance, although these results need to be confirmed for additional cultivars and *E. lata* isolates (Rolshausen *et al.*, 2006; Sosnowski *et al.*, 2022; ; Sosnowski *et al.*, 2022; Travadon *et al.*, 2024). However, these findings also suggest that selecting for current physiological resistance mechanisms could limit lesion spread but produce earlier declines in carbon gain, yield, and quality for growers. Future work should address whether selecting for alternative mechanisms (e.g. higher proanthocyanidin/catechin contents) could produce resistant cultivars that are less prone to carbon limitations.

This study is the first to compare the effects of *E. lata* on gas exchange and its photochemical and hydraulic drivers in cultivars that vary in susceptibility, and we found that infection produced opposite responses, reducing gas exchange in resistant Zinfandel and increasing it in susceptible Syrah (Tables 2, 3; Fig. 2). Previous work has shown that fungal disease can impact vine gas exchange. *Eutypa lata* was associated with a slightly higher *g*_s_ in young vines of another susceptible variety, Grenache; *Phaeomoniella chlamydospora* (Petri disease) was associated with a higher *g*_s_ in young vines of Zinfandel, Chardonnay, and Cabernet Sauvignon; and some pathogens in the Esca complex (*P. chlamydospora*, *Phaeoacremonium minimum*, and *Fomitiporia* sp.) were associated with lower *g*_s_ in mature vines of Sauvignon blanc, Chardonnay, and Cabernet Sauvignon (Petit *et al.*, 2006; Edwards *et al.*, 2007a, b; Sosnowski *et al.*, 2011; Bortolami *et al.*, 2021; Dell’Acqua *et al.*, 2024). All studies included a well-watered treatment, and it is unknown whether these differences were driven by pathogens, cultivars, or vine age. Here, gas exchange responses were more strongly determined by pathogen effects at a distance from the infection site on photosynthetic biochemistry, than effects on hydraulics at the infection site. Esca induced vines to produce xylem occlusions to compartmentalize disease spread, which can impede water transport and lower hydraulic conductivity (Bortolami *et al.*, 2019; Dell’Acqua *et al.*, 2024). However, we did not find that hydraulic conductivity was impacted by infection (Table 2), suggesting that the lower gas exchange in Zinfandel was not used to compensate for impaired hydraulic function. This could reflect methodological differences or differences between pathogens. The previous studies examined mature vines with years-long infections, allowing more time for colonization of and damage to the vasculature (Bortolami *et al.*, 2019; Dell’Acqua *et al.*, 2024). Water potentials can also change over time in stored leaves (Tomasella *et al.*, 2023), and our storage period (up to 3 d) could have contributed error to the *K*_plant_ measurements. Instead, leaf chlorophyll content and *F*_v_/*F*_m_ were higher in inoculated than in non-inoculated vines for Syrah, while *F*_v_/*F*_m_ was lower in inoculated than in non-inoculated vines for Zinfandel, consistent with the trends in gas exchange (Table 2; Fig. 3). Esca decreased photosynthesis and chlorophyll fluorescence parameters in symptomatic leaves (Petit *et al.*, 2006; Bortolami *et al.*, 2021; Dell’Acqua *et al.*, 2024), and phytotoxic metabolites produced by *E. lata* (e.g. eutypine, eulatachromene, and benzofuran) accumulate in the leaf cytoplasm and negatively impact chlorophyll content (Mahoney *et al.*, 2003; Mahoney *et al.*, 2003; Smith *et al.*, 2003). We expected Zinfandel to have better leaf detoxification strategies, such as up-regulating defensive genes that help convert toxic molecules such as eutypine to compounds that can be readily metabolized/tolerated, or genes involved in the phenylpropanoid pathway, which enhance antifungal defense by producing secondary metabolites that help plants perceive pathogens and aid in molecular crosstalk with plant stress hormones (Andolfi *et al.*, 2011; Cardot *et al.*, 2019). However, we found the opposite trend. Toxin-induced damage to the photochemical machinery could serve as a signal to close the stomata in Zinfandel (Busch, 2014), while Syrah could have used earlier detection of infection or stronger detoxification to maintain carbon assimilation to support the metabolic costs of pathogen defense. Finally, plants often accumulate osmoprotectants, which reduce oxidative stress, as part of osmotic adjustment (Yin *et al.*, 2022). Thus, we expected infection would cause plants to increase osmotic adjustment, though this has not previously been tested for grapevine trunk diseases. While osmotic adjustment occurred in both cultivars, there was no treatment effect, suggesting that osmotic adjustment does not play a pivotal role in the defense against *E. lata* infection.

Incorporating more lignin into the xylem cell walls has been suggested to increase fungal pathogen resistance by acting as a physical barrier, deterring the spread of infection and preventing rotting by reinforcing cell walls (Shigo, 1984; Rolshausen *et al.*, 2008). *Eutypa lata* produces enzymes that degrade lignin, but it preferentially degrades hemicellulose and pectin (Galarneau *et al.*, 2025). Consistent with this hypothesis, *E. lata* consumed more carbohydrates from grapevine cell walls than lignin, and the resistant cultivar Merlot had more lignin in the xylem than the susceptible cultivar Cabernet Sauvignon (Rolshausen *et al.*, 2008). However, in other studies, the relationship between wood lignin and suberin content and lesion length across cultivars was inconsistent (Munkvold and Marois, 1995), highlighting the need for more assessments of the defensive role of lignification against fungal pathogens. We expected to find a higher lignin content in Zinfandel than Syrah, but we found no significant differences between cultivars or with inoculation (Table 6). Differences in lignin content may become more pronounced as the vines mature and produce more woody biomass, and the vines in this study were 15 years younger than those tested by Rolshausen *et al.* (2008).

Phenolics are antimicrobial compounds that are typically up-regulated in response to fungal infection (Wallis and Galarneau, 2020), and their expression is associated with grapevine resistance to pathogens (Aziz *et al.*, 2020). *Eutypa lata* growth *in vitro* has been shown to be inhibited by multiple phenolic compounds, including gallic acid (a hydrolyzable tannin), rutin (a flavanol), piceid (a stilbene), and epicatechin (a proanthocyanidin/catechin) (Galarneau *et al.*, 2025). Thus, we expected infection to increase wood phenolic content, especially in Zinfandel. We found significant cultivar differences in the content of specific category classes of phenolic compounds—stilbenoids, proanthocyanidins/catechins, and other flavonoids—though not total overall phenolics. Zinfandel had higher concentrations of total proanthocyanidins/catechins, while Syrah had higher concentrations of stilbenoids and other flavonoids (Tables 6, 7). Fungal infections have been shown to up-regulate each of these phenolic classes in woody plants (Morkunas and Ratajczak 2014; Ullah *et al.*, 2017; Galarneau *et al.*, 2021), but we did not find any infection treatment effects on wood chemistry in this study (Tables 6, 7). Proanthocyanidins, also known as condensed tannins, are polymers of flavan-3-ols, such as catechins, that are present in the bark and heartwood. Proanthocyanins can deter pathogen growth by bonding to and thickening cell walls (Rudelle *et al.*, 2005; Hanlin *et al.*, 2009; Hanlin *et al.*, 2009), and higher levels have been associated with greater resistance to fungal diseases in woody species, including greater resistance to powdery mildew in grapevine (Hanlin *et al.*, 2009; Hanlin *et al.*, 2009; Wang *et al.*, 2023). Catechin also neutralized lignin-degrading enzyme activity and reduced fungal growth for other grapevine trunk disease pathogens (Gómez *et al.*, 2016). The higher levels of proanthocyanidins in Zinfandel could account for the lower levels of other flavonoid compounds, since flavonoids are their precursors in the biosynthetic pathway and are probably being converted to proanthocyanidins at a higher rate. Stilbenoids are phytoalexins that scavenge reactive oxygen species (ROS) and have been shown to limit mycelium growth in other trunk pathogens (Amalfitano *et al.*, 2000; Lambert *et al.*, 2012). Flavonoids also exhibit antifungal and antioxidant properties when stimulated in response to fungal attack (Morkunas and Ratajczak, 2014).

Our results suggest that a higher constitutive expression of proanthocyanidins and catechins could reduce pathogen spread and lesion length in Zinfandel. Fungal infections induce phenolic accumulation in existing cells near infection sites to compartmentalize the pathogen, so we expected to see the inoculation treatment up-regulate wood phenolics despite limited stem growth over the short (7 month) post-inoculation period. Instead, this period could have been too long to see infection induction effects. Previous studies showing induction measured wood phenolic content within 1 week to 3 months of fungal inoculation (Barry *et al.*, 2002; Miranda *et al.*, 2007; Lambert *et al.*, 2012; Hammerbacher *et al.*, 2014; Nemesio-Gorriz *et al.*, 2016; Wallis and Galarneau, 2020, and references within), while measurements in grape over a 3 month period found that the content of most phenolics peaked 2 months after *E. lata* inoculation (Galarneau *et al.*, 2021). Phenolics could return to baseline levels as other defense mechanisms take precedence, or the growth of new tissues farther away from the infection site with lower phenolic levels could reduce the overall wood phenolic content.

To conclude, we found that resisting damage to physiological function from *E. lata* did not increase resistance to pathogen spread, contrary to our hypotheses. Syrah exhibited longer lesion lengths, but greater levels of certain wood antifungal compounds and higher gas exchange rates and photochemical function. This could indicate that Zinfandel leaves are more vulnerable to fungal toxins, but that this vulnerability protects the woody tissues by reducing transpiration and, consequently, pathogen spread and resource delivery to the pathogen. However, this study focused on two cultivars and one *E. lata* strain, and future work should confirm that this response is a general mechanism for *Eutypa* resistance across more cultivars and pathogen strains. This study also focuses on responses 6–9 months after inoculation, and additional studies are needed to understand how short- and long-term physiological and chemical defense strategies differ. Our findings could potentially be applied in breeding for *Eutypa* resistance, since screening new plant material for infection responses in chlorophyll content and fluorescence or *g*_s_ would be faster and higher throughput than measuring lesion length. However, our findings also suggest that current physiological resistance mechanisms are not ideal for growers, who need cultivars that can both compartmentalize infection spread and maintain enough photosynthesis to avoid reductions in yield and wine quality. Thus, future work should explore whether selecting for other resistance mechanisms, such as higher constitutive proanthocyanidin/catechin levels, could reduce dependence on down-regulating gas exchange and produce cultivars that prevent lesion spread without becoming severely carbon limited. Finally, while our study focused on these traits in well-watered conditions to isolate responses to infection, future work should incorporate multiple abiotic stressors (e.g. heat and water stress) to evaluate how resistance mechanisms interact with climate. Ultimately, understanding interactions between abiotic and biotic stress responses will advance the development of more climate- and disease-resilient grape cultivars.

## Supplementary data

The following supplementary data are available at *JXB* online.

Tables S1–S5. Type III ANOVA results for qPCR-verified *Eutypa lata*-infected and mock-inoculated vines.

Table S6. Canopy area and root biomass.

Table S7. Type III ANOVA results for all wood chemistry data.

Fig. S1 and S2. Graphs of greenhouse environmental conditions during the experimental period.

Fig. S3. LICOR leaf evapotranspiration measurements.

Fig. S4. Relative water content expressed as pot water content percentage during the experimental period.

eraf103_suppl_Supplementary_Figures_S1-S4_Tables_S1-S7

## Data Availability

The data underlying this article will be shared on reasonable request to the corresponding author.
