# Supplementary material for: Differential physiological responses of resistant and susceptible grape cultivars to Eutypa dieback
Source: J Exp Bot. 2025 Mar 18;76(11):3172–85. doi: 10.1093/jxb/eraf103 (PMC12321739; doi:10.1093/jxb/eraf103)

Supplementary Materials:

Supplementary Table S1. Root biomass in grams and mean total leaf canopy area in cm^3 per cultivar per treatment. Values are means +/- standard errors. Letters show Tukey HSD test results. Post-hoc tests indicated no significant difference between cultivars or treatment.

Treatment	***Canopy Area*** **(Cm^3)**	***Root Biomass*** **(g)**
**Zinfandel (NIW)**	6950.146 ± 782.22^b	79.2 ± 20.03^a
**Zinfandel (Inoculated)**	7032.198 ± 982.25^b	68.19 ± 16.45^a
**Syrah (NIW)**	11392.410 ± 284.05^a	76.60 ± 10.35^a
**Syrah (Inoculated)**	11758.142 ± 843.97^a	87.48 ± 8.66^a

Supplementary Table S2. Type III ANOVA results for all wood chemistry data measured at the end of the experimental period with the predictors: Cultivar, Treatment, and the interaction of Cultivar x Treatment. Values are ANOVA *P*-value and boldened numbers represent significance. There were significant cultivar differences in chemistry, but no effects of treatment or interactive effects between treatment and cultivar.

**Predictor**	**Lignin**	**Total Phenolics**	**Total Catechins Procyanidins**	**Total Other Flavonoids**	**Total Stilbenoids**	
**Cultivar**	0.75	0.20	**0.0000576**	**0.00000912**	**0.007259001**	
**Treatment**	0.41	0.84	0.96	0.69	0.40	
**Cultivar x Treatment**	0.36	0.86	0.92	0.78	0.99	
	**Caftaric acid**	**Catechin**	**Epicatechin**	**Epicatechin gallate**		
**Cultivar**	**0.01637342**	**0.019772455**	**0.002730908**	**0.001532407**		
**Treatment**	0.90	0.74	0.73	0.75		
**Cultivar x Treatment**	0.57	0.75	0.77	0.81		
	**Procyanidin trimer gallate**	**Procyanidin B3**	**Procyanidin B1**	**Procyanidin B2**	**Procyanidin B2 gallate**	**Procyanidin C1**
**Cultivar**	0.75	**0.000026**	**0.0000251**	**0.00000172**	**0.0000002**	0.83
**Treatment**	0.98	0.99	0.80	0.83	0.82	0.81
**Cultivar x Treatment**	0.75	0.75	0.25	1.00	0.77	0.98
	**Unknown flavonoid glycoside**	**Kaempferol 7 O glucoside**	**Rutin**	**Quercetin glucuronide**	**Piceid**	
**Cultivar**	0.06	**0.006131269**	0.20	**0.00000309**	**0.0001115**	
**Treatment**	0.83	0.54	0.67	0.70	0.58	
**Cultivar x Treatment**	0.55	0.75	0.95	0.78	0.73	
	**Pallidol**	**Vitisin B**	**Resveratrol**	**Episilon viniferin**	**Delta viniferin**	
**Cultivar**	0.79	**0.000112586**	**0.021482809**	**0.05**	**0.0000125**	
**Treatment**	0.97	0.80	0.81	0.32	0.45	
**Cultivar x Treatment**	0.71	0.97	0.35	0.99	0.65	

SupplementaryTable S3. Type III ANOVA results for qPCR verified *Eutypalata* infected and mock-inoculated vines for variables measured once at the end of the experimental period, including lesion length, total root biomass, and canopy leaf area. (* $P<0.05$, ** $P<0.01$, *** $P<0.001$). NS represents non-significant results. Highlighted cells represent differing results in comparison to the tables presented in the main text, which uses the total dataset.

**Predictor**	**Lesion Length**	**Root biomass**	**Leaf Canopy Area**
Cultivar	*** **0.0002**	NS 0.6	*** **9.701e-05**
Treatment	NS 0.2	NS 1	NS 0.7
Cultivar x Treatment	NS 0.7	NS 1	NS 0.22

Supplementary Table S4. Type III ANOVA results for qPCR verified *Eutypalata* infected and mock-inoculated vines for variables measured repeatedly over the experiment, including stomatal conductance (g_s), photosynthesis (A), leaf-level transpiration (E), water use efficiency (WUE), pre-dawn (PDLWP) and midday stem water potentials (MDSWP), whole-plant evapotranspiration (E_{tot}), and whole-plant hydraulic conductivity (K_{plant}). Predictor variables are Cultivar, Treatment (inoculated versus wounded controls), Timepoint (days since the start of the experiment), and their interactions (Cultivar × Treatment and Treatment × Timepoint). Asterisks represent significance (* P<0.05, ** P<0.01, *** P<0.001). NS represents non-significant results. Highlighted cells represent differing results in comparison to the tables presented in the main text, which uses the total dataset.

**Predictor**	***gs***	***A***	***E***	***E*$_{tot}$**	**WUE**	**PDLWP**	**MDSWP**	**K$_{plant}$**
Cultivar	* **0.0289915**	NS 0.35	NS 0.09	*** <2E-16	NS 0.1	*** **1.7425-05**	*** **3.027E-07**	NS 0.8
Treatment	NS 0.12	* 0.0188395	NS 0.1	NS 0.15	NS 0.8	NS 0.4	NS 0.13	NS 0.7
Timepoint	*** **0.0002996**	*** **4 E-08**	* 0.036305	*** <2E-16	NS 0.2	NS 0.6	*** 0.0001151	NS 0.4
Cultivar x Treatment	** **0.0014091**	*** 0.003085	** **0.002**	NS 0.1	NS 0.1	* 0.01784	NS 0.1	NS 0.6
Treatment x Timepoint	NS 0.456	NS 0.1	NS 0.5	NS 1	NS 1	NS 0.9	NS 0.3	NS 0.7

Supplementary Table S5. Type III ANOVA results for qPCR verified *Eutypa lata* infected and mock-inoculated vines for variables measured twice during the experiment, including leaf osmotic potential at full hydration (π_o), chlorophyll content (Chl), and the quantum efficiency of PSII (*Fv/Fm*). Since these variables were measured less often, time since the start of the experiment is represented with the categorical variable Date instead of the continuous variable Timepoint (Table 2). * $P<0.05$, ** $P<0.01$, *** $P<0.001$, and NS for non-significant results. There were no differences in these variables compared to the tables presented in the manuscript.

**Predictor**	π_o	**Chl**	***Fv/Fm***
Cultivar	NS<br>0.43	***<br>**0.001129**	NS<br>0.6
Treatment	NS<br>0.6	**<br>**0.009227**	NS<br>0.3
Date	***<br>**0.0004213**	**NS**<br>**0.8**	NS<br>0.6
Cultivar x Treatment	NS<br>0.2	NS<br>0.06	NS<br>0.03
Treatment x Date	NS<br>0.4	*<br>**0.02**	NS<br>0.7

Supplementary Table S6.

Type III ANOVA results for qPCR verified *Eutypalata* infected and mock-inoculated vines wood chemistry measured at the end of the experimental period. Asterisks represent significance (* *P*<0.05, ** *P*<0.01, *** *P*<0.001). NS represents non-significant results. There were no differences in wood chemistry results compared to the tables presented in the manuscript.

**Predictor**	**Lignin**	**Total Phenolics**	**Total Procyanidins/ Catechins**	**Total Stilbenoids**	**Total Flavonoids**
Cultivar	1 NS	0.3NS	**.001*** **	**0.015** **	**0.0001*** **
Treatment	0.4 NS	1 NS	0.9 NS	0.4 NS	0.8 NS
Cultivar × Treatment	0.5 NS	0.8 NS	0.9 NS	0.9 NS	0.7 NS

Supplementary Table S7. Type III ANOVA results for all qPCR verified *Eutypalata* infected and mock-inoculated wood chemistry data measured at the end of the experimental period with the predictors: Cultivar, Treatment, and the interaction of Cultivar x Treatment. Values are ANOVA*P*-value and boldened numbers represent significance.

Compound	Cultivar	Treatment	Cultivar*Treatment
Lignin	0.97	0.42	0.48
Total Phenolics	0.33	0.96	0.83
Total Catechins Procyanidins	**0.001**	0.87	0.94
Total Other Flavonoids	**0.000**	0.80	0.71
Total Stilbenoids	**0.015**	0.44	0.91
Caftaric acid	**0.034**	0.86	0.72
catechin	0.08	0.69	0.81
epicatechin	**0.017**	0.64	0.75
Epicatechin gallate	**0.012**	0.82	0.68
Procyanidin B3	**0.000**	0.92	0.56
Procyanidin B1	8.96e-05	0.85	0.16
Procyanidin B2	3.88e-05	0.81	0.79
Procyanidin B2 gallate	3.71e-05	0.78	0.82
Procyanidin C1	0.99	0.75	0.95
Procyanidin trimer gallate	0.65	0.86	0.70
Unknown flavonoid glycoside	0.17	0.91	0.45
Kaempferol 7 O glucoside	**0.013**	0.60	0.80
Rutin	0.18	0.70	0.86
Quercetin glucuronide	9.75e-05	0.82	0.71
piceid	**0.001**	0.51	0.70
pallidol	0.43	0.88	0.51
Vitisin B	**0.001**	0.78	0.95
Resveratrol	0.06	0.65	0.38
Episilon viniferin	0.06	0.39	0.90
Delta viniferin	**0.001**	0.52	0.71

Supplementary Figures & Legends:

Supplementary Fig. S1: Greenhouse environmental conditions over the experimental period. The blue dotted line represents relative humidity and the red dotted line is temperature in degrees Celsius. The x-axis contains sampling dates: July 15th (Jul 15), August 1st (Aug 01), August 15th (Aug 15), and September 1st (Sep 01)

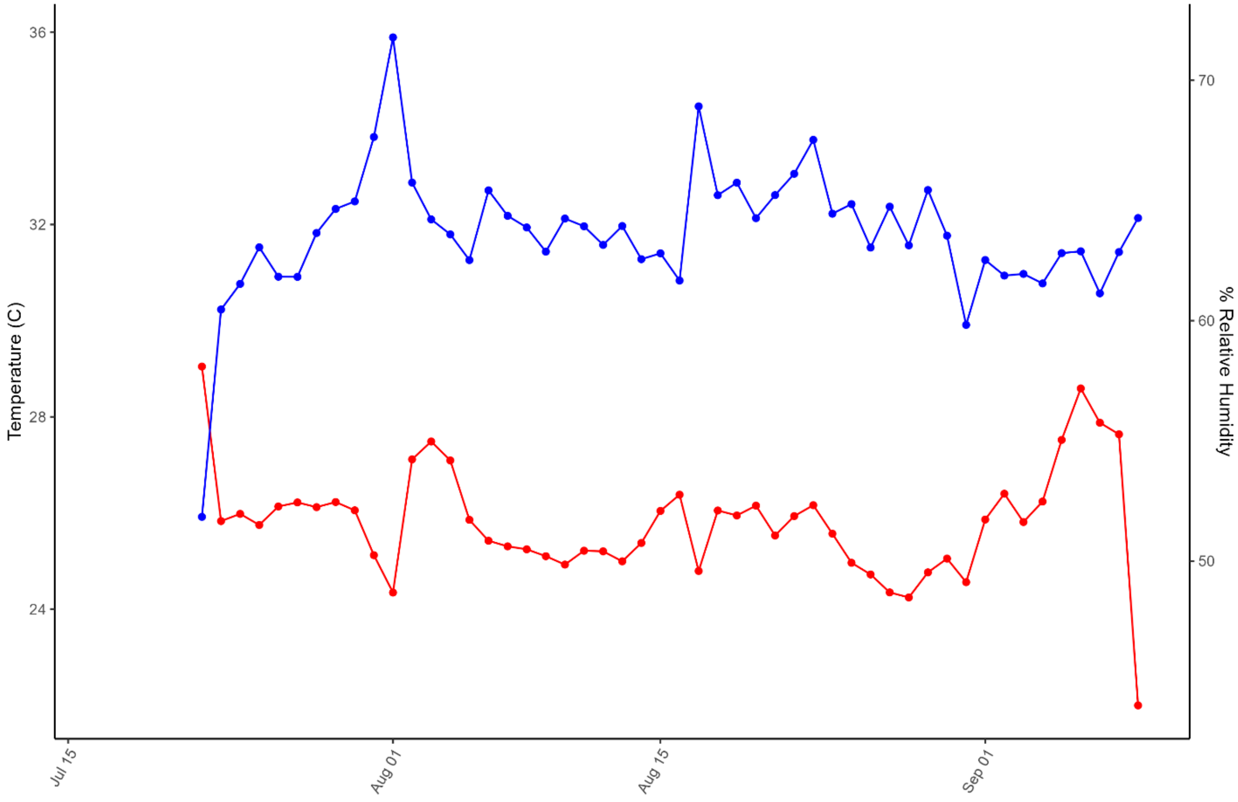

Supplementary Fig. S2: Greenhouse environmental conditions over the experimental period. The green dotted line represents VPD (kPa). The x-axis contains sampling dates: July 15th (Jul 15), August 1st (Aug 01), August 15th (Aug 15), and September 1st (Sep 01)

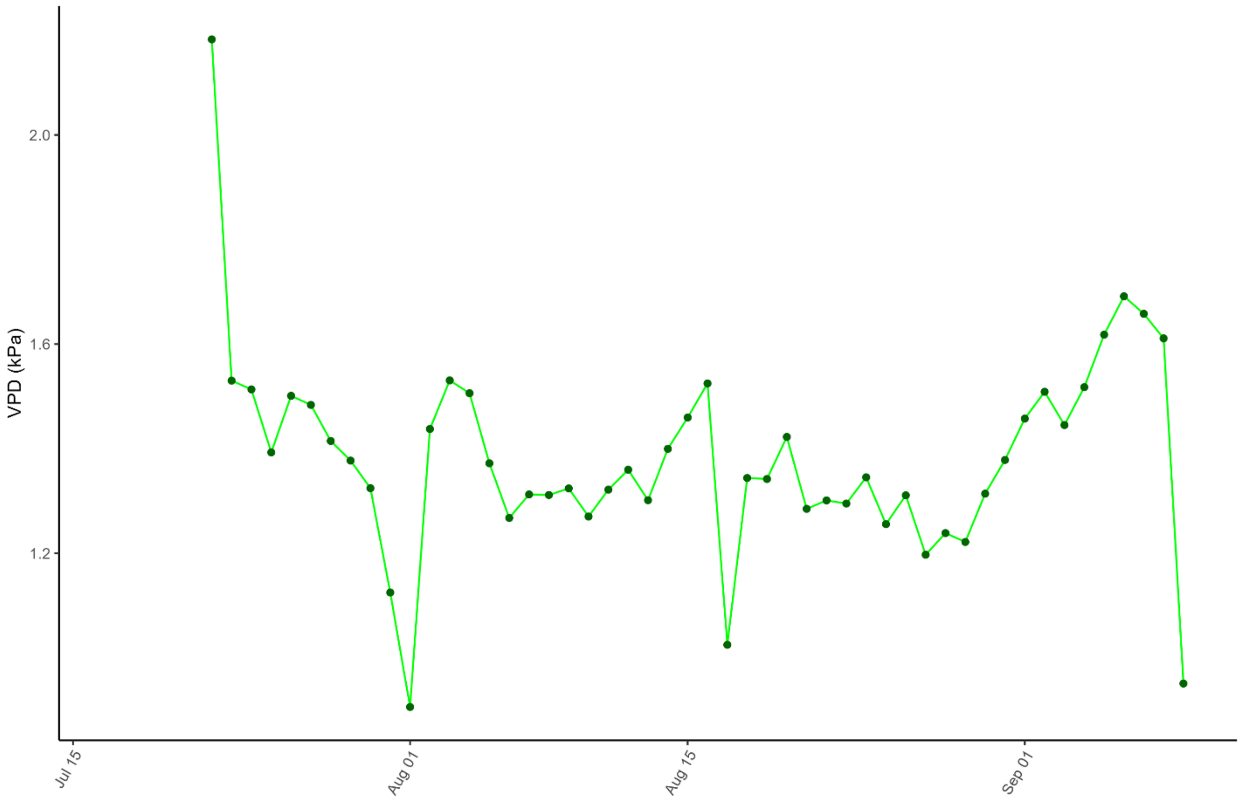

Supplementary Fig. S3: Leaf evapotranspiration (E) taken from LICOR measurements in mol m^{-2} s^{-1}. The x-axis contains sampling dates: July 15th (Jul 15), August 1st (Aug 01), August 15th (Aug 15), and September 1st (Sep 01). Data points represent averages of Syrah (SY) and Zinfandel (ZN) inoculated plants (INOC) (*N*=22-25) and Non-Inoculated Wounded control plants (NIW) (*N*= 5) per time period. All pots were under well-watered conditions.

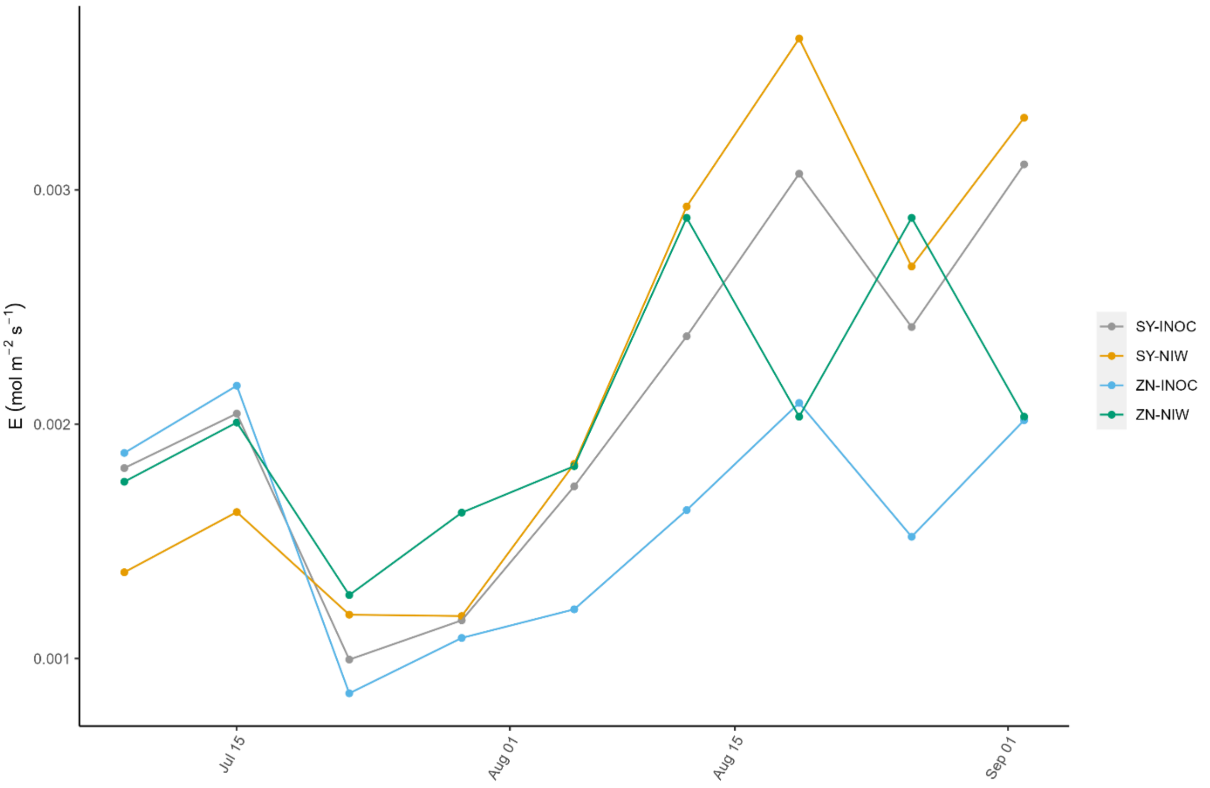

Supplementary Fig S4. (A) Relative water content expressed as pot water content percentage over the course of the experimental period for each cultivar and treatment. The x-axis contains sampling dates: July 15th (Jul 15), August 1st (Aug 01), August 15th (Aug 15), and September 1st (Sep 01). Data points represent averages of Syrah (SY) and Zinfandel (ZN) inoculated plants (INOC) (*N*=22-25) and Non-Inoculated Wounded control plants (NIW) (*N*= 5) per time period. All pots were under well-watered conditions. The pot water content was calculated as the saturated soil water content divided by the daily pot weight.
(B) Whole-plant evapotranspiration (E_{tot}) measured as the change in pot weight water loss normalized by the canopy area over the experimental period. E_{tot} is the amount of whole-plant water loss in kilograms per hour.

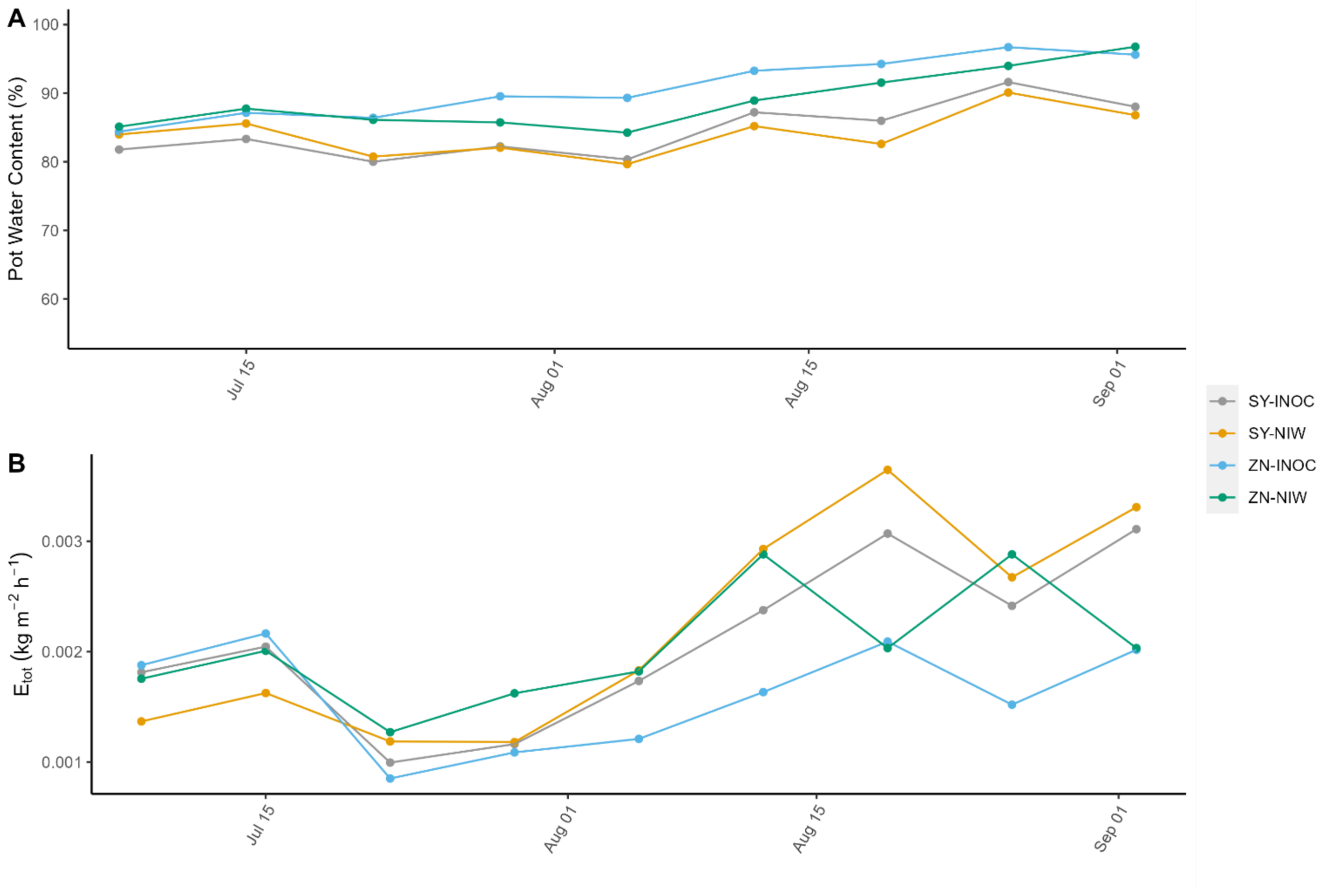

Supplement: eraf103_suppl_Supplementary_Figures_S1-S4_Tables_S1-S7 [file eraf103_suppl_supplementary_figures_s1-s4_tables_s1-s7.pdf]
